# Engineering *Zymomonas mobilis* for the Production of Xylonic Acid from Sugarcane Bagasse Hydrolysate

**DOI:** 10.3390/microorganisms9071372

**Published:** 2021-06-24

**Authors:** Christiane Ribeiro Janner Herrera, Vanessa Rodrigues Vieira, Tiago Benoliel, Clara Vida Galrão Corrêa Carneiro, Janice Lisboa De Marco, Lídia Maria Pepe de Moraes, João Ricardo Moreira de Almeida, Fernando Araripe Gonçalves Torres

**Affiliations:** 1 Departamento de Biologia Celular, Universidade de Brasília, Brasília 70910-900, DF, Brazil; chrisrjanner@gmail.com (C.R.J.H.); nessa.vrv@gmail.com (V.R.V.); tbenoliel@gmail.com (T.B.); claravidac@hotmail.com (C.V.G.C.C.); janicedemarco@uol.com.br (J.L.D.M.); lmoraes@unb.br (L.M.P.d.M.); 2 Laboratório de Genética e Biotecnologia, Parque Estação Biológica, Embrapa, Agroenergia, W3 Norte, Brasília 70770-901, DF, Brazil; joao.almeida@embrapa.br

**Keywords:** xylose, lignocellulosic biomass, xylonic acid, *Zymomonas mobilis*

## Abstract

Sugarcane bagasse is an agricultural residue rich in xylose, which may be used as a feedstock for the production of high-value-added chemicals, such as xylonic acid, an organic acid listed as one of the top 30 value-added chemicals on a NREL report. Here, *Zymomonas mobilis* was engineered for the first time to produce xylonic acid from sugarcane bagasse hydrolysate. Seven coding genes for xylose dehydrogenase (XDH) were tested. The expression of XDH gene from *Paraburkholderia xenovorans* allowed the highest production of xylonic acid (26.17 ± 0.58 g L^−1^) from 50 g L^−1^ xylose in shake flasks, with a productivity of 1.85 ± 0.06 g L^−1^ h^−1^ and a yield of 1.04 ± 0.04 g_AX_/g_X._ Deletion of the xylose reductase gene further increased the production of xylonic acid to 56.44 ± 1.93 g L^−1^ from 54.27 ± 0.26 g L^−1^ xylose in a bioreactor. Strain performance was also evaluated in sugarcane bagasse hydrolysate as a cheap feedstock, which resulted in the production of 11.13 g L^−1^ xylonic acid from 10 g L^−1^ xylose. The results show that *Z. mobilis* may be regarded as a potential platform for the production of organic acids from cheap lignocellulosic biomass in the context of biorefineries.

## 1. Introduction

To be able to change the economy from a fossil oil-based to a bio-based one, it is of utmost importance to achieve the harnessing of the entire potential of the lignocellulosic biomass [1], since it is the only carbon-rich material source available on earth other than fossils [2]. The sustainable processing of biomass in the context of biorefinery would lead to the productions of a wide range of products [3,4], including chemicals. The carbohydrate portion of lignocellulosic biomass consists predominately of the hexose sugar glucose, being the amount of xylose present in the hemicellulosic fraction highly dependent on the type of lignocellulosic material. For instance, xylose makes up approximately 23% of carbohydrates present in the sugarcane bagasse [5]. The utilization of this pentose is highly desirable for biomass valorization. Moreover, the separation of the hemicellulosic hydrolysate from the cellulosic fraction may be advantageous for an efficient conversion of xylose into valuable compounds.

Xylose has been widely considered in the production of ethanol [6,7,8,9,10,11], but it can also be used in the production of other high-value products such as xylitol [12] and levulinic acid [13]. It may also be in the production of xylonic acid, an organic acid listed as one of the top 30 value-added chemicals on a NREL report [14]. Sixteen of these chemicals are carboxylic acids; this underlines their importance as building blocks in the chemical industry, and its production from renewable sources is also possible [15].

Xylonic acid has a wide range of applications. In the pharmaceutical and cosmetic industries, it can be used as an additive in antiaging and skin renovation products to enhance skin penetration [16], as an antimicrobial agent [17] and in adsorption and retention of vitamin C [18]. Xylonic acid is also a 1,2,4-butanotriol precursor [19] and can be used as dispersant in cement [20], as well as a green solvent and catalyst for organic reactions [21]. In general, it is an alternative to gluconic acid, having the advantage of not competing with the food industry [22].

The microbial production of xylonic acid has been known since the 19th century [23], but it was only in the 1980′s that it attracted interest due to its similarity to gluconic acid along with the possibility of using cheap lignocellulosic biomass as raw material for its production. A comparison of *Pseudomonas fragi* and *Gluconobacter oxydans* strains for native production of xylonic acid from hemicellulosic biomass showed that the latter was more efficient due to its better tolerance towards inhibitors present in hemicellulose hydrolysates [24]. Besides *G. oxydans*, *Paraburkholderia sacchari* is also a native producer of xylonic acid, and, more recently, volumetric productivities as high as 4.69 g L^−1^ h^−1^ [25] and 7.7 g L^−1^ h^−1^ [26] were achieved by *G. oxydans* and *P. sacchari*, respectively. This native production of xylonic acid is possible due to the presence of an alternative xylose oxidation pathway discovered in *P. fragi* [27]. In this pathway, xylose is converted into xylonolactone by xylose dehydrogenase (XDH), which in turn is converted into xylonate by xylonolactonase (XL), even though xylonolactone can also undergo spontaneous hydrolysis to form xylonate. The pathway continues for three more steps to form α-ketoglutarate, an intermediate of the tricarboxylic acid cycle [28].

Other microorganisms were also engineered to produce xylonic acid by the heterologous expression of XDH genes with or without co-expression of XL. The yeasts *Saccharomyces cerevisiae* [29,30] and *Kluyveromyces lactis* [31], as well as the bacteria *Escherichia coli* [32,33] and *Corynebacterium glutamicum* [34], were modified in such a way. In *E. coli*, for instance, a metabolic engineering approach using programmable switches resulted in productivities up to 7.12 g L^−1^ h^−1^ [35], showing the potential of procaryotic systems for xylonic acid production. In these studies, the XDH genes employed were either *xyd1* from *Trichoderma reesei* or *xylB* from *Caulobacter crescentus*. Bioprocess development may need the search of new XDH genes which could ultimately lead to more efficient strains [36].

*Zymomonas mobilis* is a facultative anaerobe bacterium that aroused interest in the industrial production of ethanol and other value-added products. It utilizes the Entner-Doudoroff pathway, which allows fermentation with 50% less ATP production than Embden–Meyerhof–Parnas pathway, leads to a lower biomass yield and a higher uptake rate of sugars when compared to yeasts [37]. A high level production of native organic acids has been achieved in *Z. mobilis* [38] and strains with tolerance to inhibitors found in biomass hydrolysates have been isolated [37], others being engineered for the same purpose [39]. The tolerance to inhibitors is an important phenotype when utilizing lignocellulosic biomass hydrolysate, since its pre-treatment generate molecules such as furfural and 5-hydroxymethylfurfural as products of sugar degradation, being acetic acid ubiquitous in this material [40].

Given these features, we sought in this work the engineering of *Z. mobilis* for the production of xylonic acid from sugarcane bagasse hydrolysate as an inexpensive lignocellulosic feedstock. Using a rich medium, *Z. mobilis* achieved a productivity as high as 1.85 ± 0.06 g L^−1^ h^−1^ and a yield of 1.04 ± 0.04 g_AX_/g_X._ When sugarcane bagasse hydrolysate was used as a cheap feedstock, *Z. mobilis* was able to produce 11.13 g L^−1^ xylonic acid from 10 g L^−1^ xylose, showing that this bacterium may be regarded as a potential platform for the production of organic acids from lignocellulosic biomass.

## 2. Materials and Methods

### 2.1. Strains and Growth Conditions

*Z. mobilis* ZM4 (ATCC 31821) was used in this work as the host for xylonic acid production. It was routinely grown in regular RM medium (20 g L^−1^ glucose, 10 g L^−1^ yeast extract, 2 g L^−1^ potassium phosphate monobasic, 1 g L^−1^ ammonium sulphate and 1 g L^−1^ magnesium sulphate) at 30 °C. For solid medium, 15 g L^−1^ agar was added. The initial pH of the medium was 6.0.

*E. coli* strains DH5α and XL10-Gold were utilized for cloning purposes. Cells were routinely grown in an LB medium (10 g L^−1^ peptone, 10 g L^−1^ NaCl and 5 g L^−1^ yeast extract) at 37 °C. For a solid medium, 15 g L^−1^ agar was added. After transformation, cells were plated in solid medium containing the appropriated antibiotic (30 μg mL^−1^ chloramphenicol or 50 μg mL^−1^ spectinomycin). All plasmids were amplified in *E. coli* JM110 in order to demethylate the DNA prior to *Z. mobilis* transformation.

### 2.2. Strains Construction

#### 2.2.1. Strains Overexpressing XDH

Putative genes encoding for XDH were identified previously through the construction of phylogenetic trees [36]. In this work we expressed seven XDH genes which were named with a 2 letter code: CC (*xylB* from *C. crescentus*—accession # WP_010918706.1), TR (*xyd1* from *T. reesei*—accession # XP_006961719.1), BS (*Brevundimonas subvibrioides*—accession # WP_013270350.1), HL (*Halomonas lutea*—accession # WP_019016846.1), AP (SAR116 cluster alpha proteobacterium HIMB100—accession # WP_009605364.1), BX (*Paraburkholderia xenovorans*—accession # ABE37211.1) and TM (*Phaeoacremonium minimum*—accession # XP_007915223.1).

Genes encoding XDH were synthesized de novo and cloned into pMA-T (Epoch Biosciences). Specific genes were amplified from these plasmids and cloned as NdeI-BglII fragments by using In-Fusion HD EcoDry (Takara Bio Inc., Shiga, Japan) or NEBuilder HiFi DNA Assembly (New England Biolabs, Ipswich, MA, United States of America) into vector pBBR1MSC1 (chloramphenicol-resistance marker) [41], which was previously modified to contain the promotor and transcriptional termination regions from the *pdc* gene from *Z. mobilis* [42]. Plasmids constructed in this study were called pB1-XDH followed by the name of the cloned XDH gene. Primers used for these amplifications are listed in Table S1.

Gene BX was also co-expressed with XL from *P. xenovorans* (KEGG entry Bxe_C1362) in *Z. mobilis*. To achieve this, XL was synthesized de novo by Integrated DNA Technologies (IDT—Coralville, IA, USA) as a linearized DNA fragment and cloned into pB1-XDH BX digested with BglII in order to form an operon controlled by the *pdc* promoter. The resulting plasmid was called pB1-BXL.

*Z. mobilis* was transformed by electroporation. Briefly, cells were grown until an OD_600_ of 0.3 and washed twice with 10% glycerol. Cells were electroporated in a 0.2 cm cuvette using the following parameters: 2.5 kV, 200 Ω and 25 μF. After the pulse, cells were recovered by adding 900 µL RM medium following incubation at 30 °C with agitation of 600 rpm in a dry bath incubator for 4 to 18 h. Once recovered, cells were plated in solid RM medium with the appropriated antibiotic (100 μg mL^−1^ chloramphenicol or 200 μg mL^−1^ spectinomycin) for selection of transformants. Plates were incubated at 30 °C for 48 to 96 h. Table 1 shows the strains overexpressing XDH.

#### 2.2.2. Construction of Gene Deletion Cassettes and Marker Excision

Gene deletion in *Z. mobilis* was carried out by homologous recombination. Target genes were ZMO_RS04375 (old locus tag ZMO0976, an aldo/keto reductase) and ZMO_RS05565 (old locus tag ZMO1237, a D-lactate dehydrogenase). Primers used for construction of gene deletion cassettes and to confirm events of integration and marker excision are listed in Table S1. Briefly, upstream and downstream sequences of ~500 bp flanking the coding regions of each gene to be deleted were amplified from the *Z. mobilis* genome. Deletion cassettes were constructed in vitro by using the NEBuilder HiFi DNA Assembly protocol (NEB). Reactions consisted of 5′-upstream and 3’-downstream amplicons, a spectinomycin resistance marker flanked by *loxP* sites and the cloning vector pPCV [43] linearized with KpnI and SacI. After amplification in *E. coli*, the resulting vectors were used to transform *Z. mobilis* following selection on plates containing spectinomycin. Gene deletion was confirmed by PCR. In order to excise the spectinomycin marker, the Cre recombinase gene was heterologously expressed. This gene was amplified from pYRCre2 vector [44] and cloned as a NdeI-BglII fragment into pBBR1MSC1 [41] as described previously. The resulting vector was transformed into the knockout strains. A single colony was streaked on RM plates with no antibiotic in order to obtain isolated colonies. Single colonies were individually transferred to three separated plates containing chloramphenicol, spectinomycin or no antibiotics. Colonies that grew only in medium without antibiotics were submitted to genomic PCR for confirmation. All strains constructed in this work are presented in Table 1.

### 2.3. Strain Selection

For the initial screening for XDH activity, fermentations were performed in 250 mL Erlenmeyer flasks containing 25 mL modified RM 1 (RM supplied with 5 g L^−1^ glucose, 50 g L^−1^ xylose, which was defined in this work). Cells were first grown in 100 mL of regular RM and then inoculated at OD_600_ of 7.0 in modified RM. The high initial cell concentration was used seeking improvement in volumetric productivities and evaluate XDH activity. Xylonic acid production was carried out at 30 °C and 200 rpm. The deleted strains and the strain co-expressing BX and XL were evaluated in the same way. All tests were performed in triplicates.

### 2.4. Xylonic Acid Production

A selected strain was grown in regular RM and inoculated at an OD_600_ = of 1.0 in a 1 L-scale laboratory bioreactor (Multifors 2, Infors AG, Basel, Switzerland) containing 500 mL modified RM 2 (RM supplied with 20 g L^−1^ glucose, 50 g L^−1^ xylose) at 30 °C, pH 6.0, stirring speed at 200 rpm and airflow at 0.3 vvm. The medium and conditions were defined in this work. The pH was maintained by addition of 3 M KOH and the experiment was performed in duplicates.

To investigate the ability of using lignocellulosic hydrolysate in the xylonic acid production by *Z. mobilis*, another fermentation was performed using C5 hydrolysate from sugarcane bagasse (8.5 g L^−1^ glucose, 100 g L^−1^ xylose, 20 g L^−1^ acetate, 1 g L^−1^ 5-hydroxymetilfurfural and 2 g L^−1^ furfural), which was added at 10% in defined medium (2 g L^−1^ potassium phosphate monobasic, 1 g L^−1^ ammonium sulphate, 1 g L^−1^ magnesium sulphate, 0.2 g L^−1^ calcium chloride, 25 mg L^−1^ sodium molybdate, 25 mg L^−1^ ferrous sulphate, 0.05 mg L^−1^ calcium pantothenate, 0.05 mg L^−1^ thiamine, 0.02 mg L^−1^ biotin and 0.05 mg L^−1^ nicotinic acid). Glucose was supplemented to a final concentration of 20 g L^−1^. The fermentation was performed in a 1 L-scale laboratory bioreactor (Multifors 2) containing 500 mL of medium at 30 °C, pH 6.0, stirring speed at 200 rpm and airflow at 0.3 vvm. The pH was maintained by addition of 3 M KOH.

### 2.5. Growth Tests in Microplates

ZM4 BXL strain had its growth compared to ZM4 BX and a negative control in microplates. A fresh culture was inoculated in 2 mL of regular RM or modified RM 2 medium with appropriate antibiotic to achieve a OD_600_ = 0.15 in a 12-well plate. The culture was incubated at 30 °C and 200 rpm, and the cell growth performed in Epoch Microplate Spectrophotometer (Bio Tek Instruments Inc., Winooski, VT, United States of America) with data collection at every 30 min. This experiment was conducted in triplicates.

### 2.6. Calculation of Productivity, Yield and Specific Growth Rate

The volumetric productivity was calculated by plotting the production of xylonic acid versus the culture time and the slope of the resulting linear regression was assumed as the volumetric productivity. Similarly, the yield is equal to the slope obtained by performing a linear regression of the plot of the production of xylonic acid versus the consumed xylose. The specific growth rate was calculated by plotting the logarithm of the cell density versus the culture time during the exponential growth phase and the slope of the linear regression was considered as the maximum specific growth rate.

### 2.7. Analytical Methods

Extracellular glucose, xylose and xylonic acid were analyzed by HPLC (LC-20A Prominence—Shimadzu) equipped with a Rezex ROA-Organic Acid (300 × 7.8 mm) column maintained at 55 °C and using 5 mM H_2_SO_4_ as mobile phase at a flow rate of 0.4 mL min^−1^ [32]. Glucose and xylose peaks were detected with a refractive index detector (RID-10A), whereas xylonic acid peaks were detected and quantified from an UV/VIS detector (SPD-20A). Once xylonic acid and xylose have the same retention time, when the former was present the latter was indirectly quantified by subtracting the xylonic acid peak (quantified by UV) from the combined xylose and xylonic acid peaks detected by RID [34].

## 3. Results and Discussion

### 3.1. Xylonic Acid Production by Z. mobilis Expressing XDH Genes

*Z. mobilis* has the potential to establish a novel platform for the production of different molecules of biotechnological interest, playing a critical role in the replacements of petrochemical products [45]. This includes the production of aldonic acids, such as xylonic acid, due to the presence of the enzyme glucose-fructose oxidoreductase (GFOR), which is responsible for the conversion of fructose and glucose into sorbitol and gluconolactone, respectively [46]. GFOR also shows affinity for xylose, converting 42% of this sugar to xylonic acid in the presence of fructose [47]. However, GFOR has a disadvantage due to its requirement of a co-substrate, fructose, which is expensive and would lead to by-product formation [47]. Alternatively, the heterologous expression of a xylose dehydrogenase gene may be accomplished for the same purpose.

**Figure 1:**
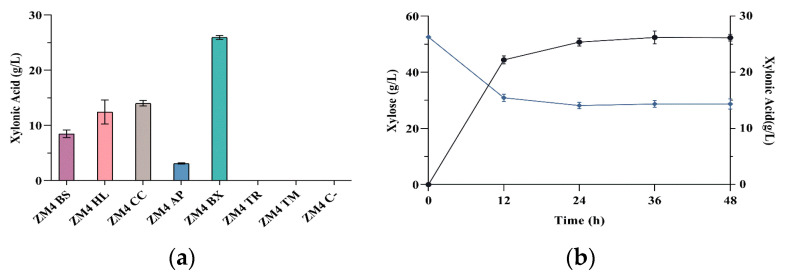
(**a**) Production of xylonic acid by *Z. mobilis* strains expressing different XDH genes from the following microorganisms: *Brevundimonas subvibrioides* (BS); *Halomonas lutea* (HL); *Caulobacter crescentus* (CC); SAR116 cluster alpha proteobacterium HIMB100 (AP); *Paraburkholderia xenovorans* (BX); *Trichoderma reesei* (TR) and *Phaeoacremonium minimum* (TM). Fermentation was performed in shake flasks for 48 h at 30 °C in modified RM 1 medium containing 50 g L^−1^ xylose. (**b**) Time course production of xylonic acid (dark blue circles) and xylose consumption (light blue diamonds) of ZM4 BX. The strain was grown in shake flasks with 50 g L^−1^ xylose at 30 °C.

To evaluate the ability of *Z. mobilis* to produce xylonic acid from XDH, seven different genes were overexpressed in this bacterium (Table 1), including a control (CC) from *C. crescentus* which is widely used in microbial metabolic engineering for the production of this organic acid. The seven strains were cultivated in a modified RM medium containing 5 g L^−1^ glucose as a carbon source and 50 g L^−1^ xylose as the substrate for xylonic acid production. The efficiencies of these strains towards the production of xylonic acid after 48 h of cultivation in shake flasks are shown in Figure 1a; Table 2 shows the production and productivity achieved by each strain in this experiment. The expression of five different prokaryotic XDH genes resulted in different levels of xylonic acid production. Interestingly, *Z. mobilis* strains expressing the two eukaryotic XDH genes, TR and TM, did not produce detectable amounts of xylonic acid, hence they were not further used in this work. Remarkably, strain ZM4 BX, which carries the XDH gene from *P. xenovorans*, showed a ~2-fold improvement in xylonic acid production when compared to the control ZM4 CC. In this particular experiment, ZM4 BX produced 26.17 ± 0.58 g L^−1^ xylonic acid with a yield of 1.04 ± 0.04 g_AX_/g_X_, which represents 93.7% of the maximum theoretical yield, showing that almost all consumed xylose is being converted into xylonic acid. In this experiment, after 24 h growth, there was no significant production of xylonic acid nor consumption of xylose which is probably due to acidification, since the medium was not buffered and the final pH reached 3.00 ± 0.13 (Figure 1b).

It is noteworthy that the productivity of 1.85 ± 0.06 g L^−1^ h^−1^ presented by ZM4 BX is higher than that observed for other engineered microorganisms described in the literature. By comparing volumetric productivities, ZM4 BX is amongst the best engineered strains for xylonic acid production. For instance, when the yeast *S. cerevisiae* was engineered to produce xylonic acid, it achieved productivities of 36 mg L^−1^ h^−1^ [29] and 0.24 g L^−1^ h^−1^ [30] when expressing *xyd1* from *T. reesei* or co-expressed *xylB* and *xylC* from *C. crescentus*, respectively. Bacteria such as *E. coli* and *C. glutamicum* were engineered to xylonic acid production as well. An *E. coli* strain expressing *xylB* from *C. crescentus* achieved a productivity of 1.09 g L^−1^ h^−1^ [32], and strains of *C. glutamicum* expressing the same gene achieved productivities of 1.02 g L^−1^ h^−1^ [34] and 0.93 g L^−1^ h^−1^ [48]. A similar value (productivity of 1.8 g L^−1^ h^−1^) was also encountered for two *E. coli* strains engineered to produce xylonic acid [33,49]. The best productivity described so far for engineered microorganisms was achieved by *E. coli* expressing *xylB* from *C. crescentus* and further engineered by the construction of protein circuits, leading to a productivity of 7.12 g L^−1^ h^−1^ [35]; this result underlines the use of synthetic biology approaches for strain engineering. In regard to native producers, volumetric productivities of 4.69 g L^−1^ h^−1^ [25] and 7.7 g L^−1^ h^−1^ [26] were achieved by *G. oxydans* and *P. sacchari*, respectively. Although higher than that obtained by *Z. mobilis*, this bacterium may present some advantages that can be exploited for the value-added materials production, such as the presence of an energetically uncoupled metabolism with high catabolic flux rates and the small fraction of substrate that is converted into biomass [50]. Based on the performance of ZM4 BX for xylonic acid production, this strain was chosen for further analysis.

### 3.2. Simultaneous Expression of XDH and XL Genes

Although xylonic acid production may be achieved only by expressing XDH since xylonolactone undergoes spontaneous dehydration to form this organic acid, we reasoned that the expression of XL would improve production, as previously observed in *E. coli* [33]. For this reason, the gene encoding XL from *P. xenovorans* was expressed in ZM4 BX.

**Figure 2:**
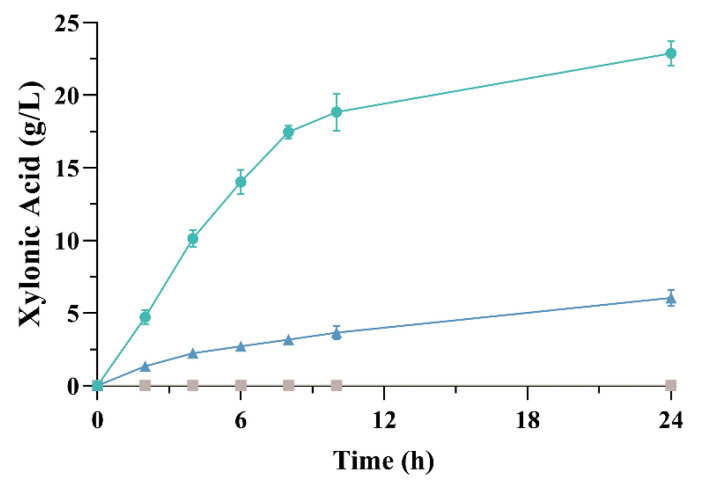
Comparative production of xylonic acid between ZM4 BX (green circles), ZM4 BXL (blue triangles) and ZM4 C- (gray squares). Strains were cultivated in shake flasks with modified RM medium containing 50 g L^−1^ xylose at 30 °C.

The strain co-expressing XDH and XL (ZM4 BXL) was evaluated for xylonic acid production. Interestingly, xylonic acid production was impaired, as shown in Figure 2. While the strain expressing only BX was able to produce 22.88 ± 0.83 g L^−1^ xylonic acid after 24 h growth, ZM4 BXL produced only 6.06 ± 0.57 g L^−1^, and the strain ZM4 C- was not able to produce any xylonic acid. A similar result was observed in a *C. glutamicum* strain expressing *xylB* (xylose dehydrogenase) and *xylC* (xylonolactonase) from *C. crescentus*, which produced 5.86 g L^−1^ xylonic acid compared to 20.04 g L^−1^ produced by the strain expressing only *xylB* [34]. Once XL catalyzes the opening of the lactone ring to the linear form xylonate, we hypothesized that the intracellular pH may have dropped rapidly. In a study with a xylonic acid producing strain of *S. cerevisiae* [51], it was demonstrated that the co-expression of *xylC* with *xylB* from *C. crescentus* indeed leads to a rapid decrease of the intracellular pH to < 5 compared to the strain expressing only *xylB*; this leads to a reduction on the specific growth rate and cell death. This would explain the poor performance of *Z. mobilis* when expressing both genes.

Aiming to confirm the decrease in the specific growth rate due to XL expression when xylose was present in the medium, strains ZM4 BX and ZM4 BXL were grown in medium containing only glucose or glucose plus xylose. The results showed that the strain expressing both genes grew less than ZM4 BX in the presence of xylose (Figure S1). When grown only in glucose, ZM4 BXL presented a better growth, with a maximum specific growth rate of 0.47 ± 0.04 h^−1^ compared to 0.38 ± 0.01 h^−1^ of ZM4 BX. When xylose was added to the medium, the maximum specific growth rate of ZM4 BXL was 0.14 ± 0.05 h^−1^, 55% less than ZM4 BX (0.31 ± 0.01 h^−1^). Given its negative effects on cell growth, XL was no longer tested in this work.

### 3.3. Inactivation of Native Competing Pathways in Z. mobilis

Metabolic engineering allows transforming microorganisms into efficient cell factories for the compounds of interest. Among the rational approaches used in metabolic engineering, we chose the removal of a competing pathway by gene deletion [52].

*Z. mobilis* contains a xylose reductase (XR) which produces xylitol from xylose [53]. The gene coding for XR was chosen for knockout since this enzyme could compete for xylose and generate an undesirable by-product which is toxic to the cells [54]. To reduce other by-products formation, ZMO1237 was also deleted. This gene codes for a lactate dehydrogenase which produces lactate from pyruvate mainly under aerobioses [55], a condition that would benefit xylonic acid formation due to the co-factor recycling by the respiratory chain. Therefore, ZMO1237 was chosen for gene knockout since lactate production from glucose metabolism could interfere in biomass formation.

Deletion cassettes were constructed in pPCV vector [43] since its origin of replication is not recognized by *Z. mobilis* and thus would function as a suicide plasmid favoring the selection of integration events. After transformation, gene deletion was confirmed by PCR and selected clones were transformed with a plasmid containing Cre-recombinase in order to excise antibiotic marker (Figure S2). Using this approach, three strains were generated bearing both genes disrupted separately or together, as shown in Table 1.

**Figure 3:**
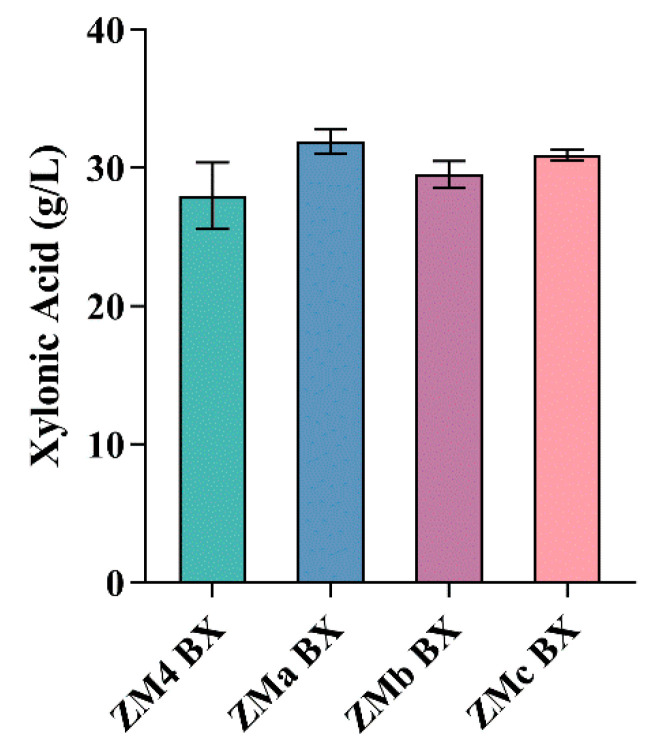
Xylonic acid production of engineered *Z. mobilis* strains expressing BX. Strains ZM4 (wild-type), ZMa (mutant for xylose reductase), ZMc (mutant for lactate dehydrogenase) and ZMc (double mutant) were transformed with plasmid pB1-XDH BX and grown for 24 h in shake flasks with modified RM 1 medium containing 50 g L^−1^ xylose at 30 °C.

These strains were transformed with pB1-XDH BX plasmid and xylonic acid production was evaluated. As shown in Figure 3 and Table 3, all tested strains produced similar amounts of xylonic acid after 24 h cultivation in xylose. Although the knockout strains showed slightly higher means of xylonic acid production, the *t*-test (*p* < 0.1) showed that only ZMa BX presented a significant difference, showing that this strain was capable of producing more xylonic acid than ZM4 BX. This difference reflects that xylose is not being converted into xylitol, a toxic compound for *Z. mobilis*. The production of xylonic acid by ZMb BX was not significantly higher than ZM4 BX, which demonstrates that disruption of the lactate dehydrogenase gene does not affects xylonic acid production by this bacterium. Although ZMc also bears a deletion of ZMO0976 as ZMa, it is possible that gene knockout of ZMO1237 caused an opposite effect, since lactate dehydrogenase recycles NAD^+^ used as a co-factor by XDH, and should not be consider a target for *Z. mobilis* engineering for xylonic acid production.

### 3.4. Production of Xylonic Acid in a Batch Fermentation

**Figure 4:**
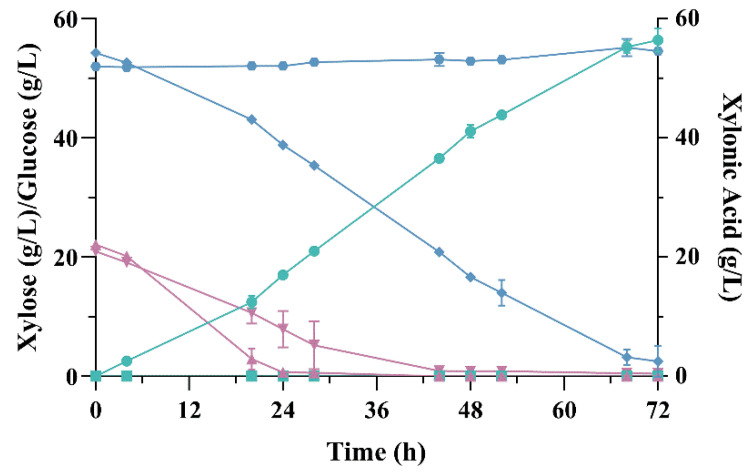
Fermentative profile of strains ZMa BX and ZMa C-. Fermentation was carried out in a bioreactor with modified RM medium containing 50 g L^−1^ xylose in batch mode at 30 °C. Xylonic acid production by ZMa BX (green circles), xylose (blue diamonds) and glucose (purple triangles) consumption by ZMa BX; xylonic acid production by ZMa C- (green squares), xylose (blue hexagons) and glucose (purple inverted triangles) consumption by ZMa C-.

To produce xylonic acid under controlled conditions, strain ZMa BX was chosen due to its better xylonic acid production as compared to ZM4 BX described above. It was grown in a 1 L-scale bioreactor containing 500 mL of modified RM 2 medium in a batch fermentation mode (Figure 4). Glucose was consumed in ~28 h and the consumption of xylose begun after 4 h, ending in ~72 h. At this point, ZMa BX produced 56.44 ± 1.93 g L^−1^ xylonic acid from 54.27 ± 0.26 g L^−1^ xylose, with a yield of 1.08 ± 0.02 g_AX_/g_X_, which represents 97.3% of the theoretical maximum. The productivity of this bioprocess was 0.99 ± 0.03 g L^−1^ h^−1^, which was smaller than that obtained in shake flasks—this is probably due to the differences in the initial concentration of biomass in each experiment.

Strain ZMa C- (negative control) was unable to utilize any xylose, which remained in the medium until the end of the fermentation. Interestingly, it also showed a slower growth rate compared to the xylonic acid producing strain, since the latter presented a specific growth rate of 0.04 ± 0.00 h^−1^ in comparison to 0.02 ± 0.00 h^−1^ of the negative control. Indeed, glucose was consumed within 52 h in comparison to 28 h for ZMa BX. This may be explained by the stress caused by xylose itself. Molecular responses to the presence of xylose have already been investigated in a *Z. mobilis* xylose-utilizing strain, showing that this pentose causes a more severe impact in this bacterium than that caused by acetate treatment, recruiting more genes for xylose utilization than it does for acetate [54]. Although being different strains, xylose may pose some stress in ZMa C- as well; further metabolic engineering studies are required to elucidate the mechanisms of xylose utilization by *Z. mobilis* in order to improve the harnessing of this sugar.

### 3.5. Use of Sugarcane Bagasse Hydrolysate in a Batch Fermentation

Sugarcane bagasse is an abundant agricultural residue in Brazil, with a production of 178 million tons in 2016 [56], which makes its utilization of utmost interest. Since *Z. mobilis* showed an efficient conversion of xylose into xylonic acid, the use of a hemicellulosic hydrolysate derived from sugarcane bagasse as an inexpensive feedstock for the production of this organic acid was pursued. This test would also represent an opportunity to assess the inhibitory effects of the hydrolysate on *Z. mobilis*.

**Figure 5:**
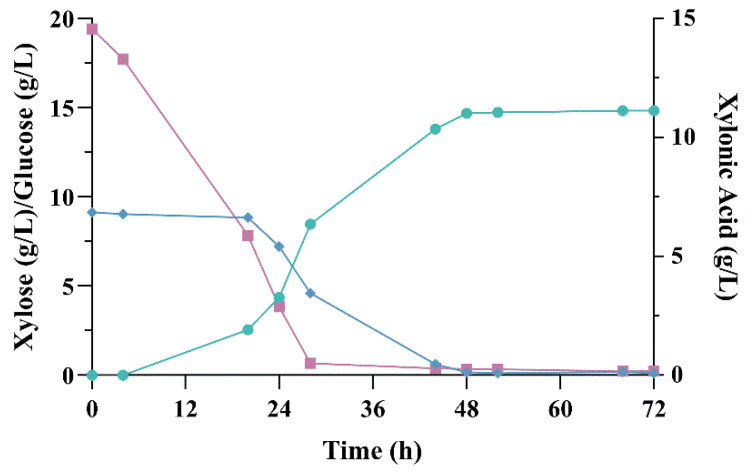
Production of xylonic acid from sugarcane bagasse hydrolysate. Fermentation was carried out in a bioreactor with defined medium containing 10% hemicellulosic hydrolysate of sugarcane bagasse in batch mode at 30 °C. Xylonic acid (green circles), xylose (blue diamonds) and glucose (purple squares).

We used a defined media containing 10% sugarcane bagasse hydrolysate which contained ~10 g L^−1^ xylose. ZMa C- strain was not able to grow nor consume glucose present in the medium, whereas ZMa BX consumed it after 28 h cultivation (Figure 5). This strain achieved a xylonic acid production of 11.13 g L^−1^ with a productivity of 0.32 g L^−1^ h^−1^, which is about one third of that obtained in rich medium, probably due to the presence of inhibitors found in lignocellulosic hydrolysate. Despite of that, the yield achieved in medium containing sugarcane bagasse hydrolysate was 1.07 g_AX_/g_X_ after 72 h growth, which was similar for that encountered in rich medium.

The productivity achieved by ZMa BX in sugarcane bagasse hydrolysate is smaller than that encountered for an engineered *E. coli* strain, which was 1.52 g L^−1^ h^−1^ [49] when a corn cob hydrolysate was used as feedstock. A modified *C. glutamicum* strain using rice straw hydrolysate as feedstock was able to produce 42.94 g L^−1^ xylonic acid from 60 g L^−1^ xylose only after 120 h incubation [48]. However, *Z. mobilis* was able to convert all xylose available in the medium containing hydrolysate into xylonic acid, showing its potential as a platform for industrial production of this organic acid.

An interesting result from this experiment was that *Z. mobilis* was able to detoxify furfural from hydrolysate, since this compound was no longer detected in the end of the fermentation (Figure S3). After 4 h of fermentation, the furfural peak in the chromatogram decreased to approximately half of its height, and the OD_600_ at this point was 1.03. Indeed, it was demonstrated that *Z. mobilis* is capable of converting furfural into its alcohol form furfuryl, which is roughly one fourth as inhibitory as furfural [57]. This is an important attribute when considering lignocellulosic hydrolysate as feedstock for production of biotechnological valuable molecules.

## 4. Conclusions

This work is the first report of an engineered *Z. mobilis* strain capable of producing xylonic acid from xylose. The engineered strain was able to convert xylose in sugarcane bagasse hydrolysate into xylonic acid, showing the potential of using *Z. mobilis* in an economically feasible and eco-friendly bioprocess. This work underlines the importance of exploring new genes derived from the biodiversity in order to improve metabolic pathways in the context of synthetic biology.

This process of xylonic acid production can be further optimized by changing and evaluating the fermentation parameters such as pH, air flow, inoculum concentration and medium. In this regard, the assessment of the need of adding vitamins, and its concentrations, when utilizing sugarcane bagasse hydrolysate is of utmost importance in the appraising of the economic viability of the process, thus, requiring further investigation.

## Figures and Tables

**Table 1:** *Z. mobilis* strains used in this work.

Strain	Genotype	Reference
ZM4	wild-type *Z. mobilis* ZM4	ATCC 31821
ZM4 CC	ZM4 containing pB1-XDH CC	This work
ZM4 BS	ZM4 containing pB1-XDH BS	This work
ZM4 HL	ZM4 containing pB1-XDH HL	This work
ZM4 AP	ZM4 containing pB1-XDH AP	This work
ZM4 BX	ZM4 containing pB1-XDH BX	This work
ZM4 TR	ZM4 containing pB1-XDH TR	This work
ZM4 TM	ZM4 containing pB1-XDH TM	This work
ZM4 BXL	ZM4 containing pB1-BXL	This work
ZM4 C-	ZM4 containing the empty vector pBBR1MCS1	This work
ZMa	ZM4, ΔZMO0976	This work
ZMb	ZM4, ΔZMO1237	This work
ZMc	ZM4 ΔZMO0976, ΔZMO1237	This work
ZMa BX	ZMa containing pB1-XDH BX	This work
ZMb BX	ZMb containing pB1-XDH BX	This work
ZMc BX	ZMc containing pB1-XDH BX	This work
ZMa C-	ZMa containing the empty vector pBBR1MCS1	This work
ZMb C-	ZMb containing the empty vector pBBR1MCS1	This work
ZMc C-	ZMc containing the empty vector pBBR1MCS1	This work

**Table 2:** Production and productivity achieved by *Z. mobilis* strains overexpressing different XDH genes.

Strain	Xylonic Acid	
	Production (g L^−1^)	Productivity (g L^−1^ h^−1^)
ZM4 CC	14.01 ± 0.70	0.96 ± 0.03
ZM4 BS	8.47 ± 0.97	0.63 ± 0.02
ZM4 HL	12.45 ± 3.05	0.88 ± 0.11
ZM4 AP	3.10 ± 0.20	0.24 ± 0.01
ZM4 BX	26.17 ± 0.58	1.85 ± 0.06
ZM4 TR	n.d.	n.d.
ZM4 TM	n.d.	n.d.
ZM4 C-	n.d.	n.d.

n.d.: not detected.

**Table 3:** Production of xylonic acid, productivity and yield achieved by engineered strains of *Z. mobilis* expressing BX after 24 h of cultivation.

Strain	Xylonic Acid		
	Production (g L^−1^)	Productivity (g L^−1^ h^−1^)	Yield (g_AX_/g_X_)
ZM4 BX	27.99 ± 2.40	2.12 ± 0.19	1.05 ± 0.01
ZMa BX	31.90 ± 0.88	2.28 ± 0.19	1.05 ± 0.06
ZMb BX	29.55 ± 0.95	2.25 ± 0.13	1.03 ± 0.11
ZMc BX	30.91 ± 0.36	2.36 ± 0.06	1.05 ± 0.06

## Data Availability

The data used and/or analyzed in this study are available on request from the corresponding author.

## Supplementary Materials

The following are available online at https://www.mdpi.com/article/10.3390/microorganisms9071372/s1, Figure S1: Growth of ZM4 BX and ZM4 BXL strains in glucose or glucose plus xylose: open triangles: ZM4 BXL on glucose; open circles: ZM4 BX on glucose; closed triangles: ZM4 BXL in glucose and xylose. closed circles: ZM4 BX on glucose and xylose, Figure S2: Deletion of genes ZMO0976 and ZMO1237 in *Z. mobilis*. After transformation, gene deletion and marker excision were assessed by PCR with specific primers. A: Deletion of ZMO0976 (confirmation primers U-ZMO0976-F/D-ZMO0976-R). M1: 1 kb Plus DNA Ladder (Invitrogen), WT: wild-type ZMO0976 (~3 kb); Δ0976: ΔZMO0976::*Spc^R^* (~3.3 kb); ZMa: ΔZMO0976 (~2.1 kb). B: Deletion of ZMO1237 (confirmation primers Cldh-F/Cldh-R). M2: 1 kb DNA Ladder (Promega); WT: wild-type ZMO1237 (~2.2 kb); Δ1237: ΔZMO1237::*Spc^R^* (~2.5 kb); ZMb: ΔZMO1237 (~1.3 kb). C: Deletion of ZMO1237 in ZMa strain. M1: 1 kb Plus DNA Ladder (Invitrogen); ZMa: ΔZMO0976—wild-type ZMO1237 (~2.2 kb); Δ1237: ΔZMO0976, ΔZMO1237::*Spc^R^* (~2.5 kb, primers Cldh-F/Cldh-R); ZMc: ΔZMO0976, ΔZMO1237 (~1.3 kb, primers Cldh-F/Cldh-R). *Spc^R^*: spectinomycin resistance cassette. C-: reaction control, Figure S3: Stacked chromatograms showing the decrease in furfural peak (retention time ~70 min). Standard containing furfural (1); fermentation sample at time 0 h (2); fermentation sample after 4 h (3); fermentation sample after 20 h (4).; Table S1: Primers used for amplification of XDH genes, construction of deletion cassettes, knockout confirmation and Cre recombinase amplification.
