# Supplementary material for: Engineering Zymomonas mobilis for the Production of Xylonic Acid from Sugarcane Bagasse Hydrolysate
Source: Microorganisms. 2021 Jun 24;9(7):1372. doi: 10.3390/microorganisms9071372 (PMC8304316; doi:10.3390/microorganisms9071372)
Supplement: Supplementary file 1 [file microorganisms-09-01372-s001.zip › microorganisms-1246379-supplementary.pdf]

Supplementary material

Engineering *Zymomonas mobilis* for the production of xylonic acid from sugarcane bagasse

Christiane Ribeiro Janner Herrera[1], Vanessa Rodrigues Vieira[1], Tiago Benoliel[1], Janice Lisboa De Marco[1], Clara Vida Galvão Corrêa Carneiro[1,2], Lídia Maria Pepe de Moraes[1], João Ricardo Moreira de Almeida[2], Fernando Araripe Gonçalves Torres[1]

[1]Departamento de Biologia Celular, Universidade de Brasília, Brasília, DF, 70910-900, Brazil

[2]Embrapa Agroenergia, Laboratório de Genética e Biotecnologia, Parque Estação Biológica, W3 Norte, Brasília, DF, 70770-901, Brazil

Corresponding author: F. A. G. Torres

E-mail address: ftorres@unb.br

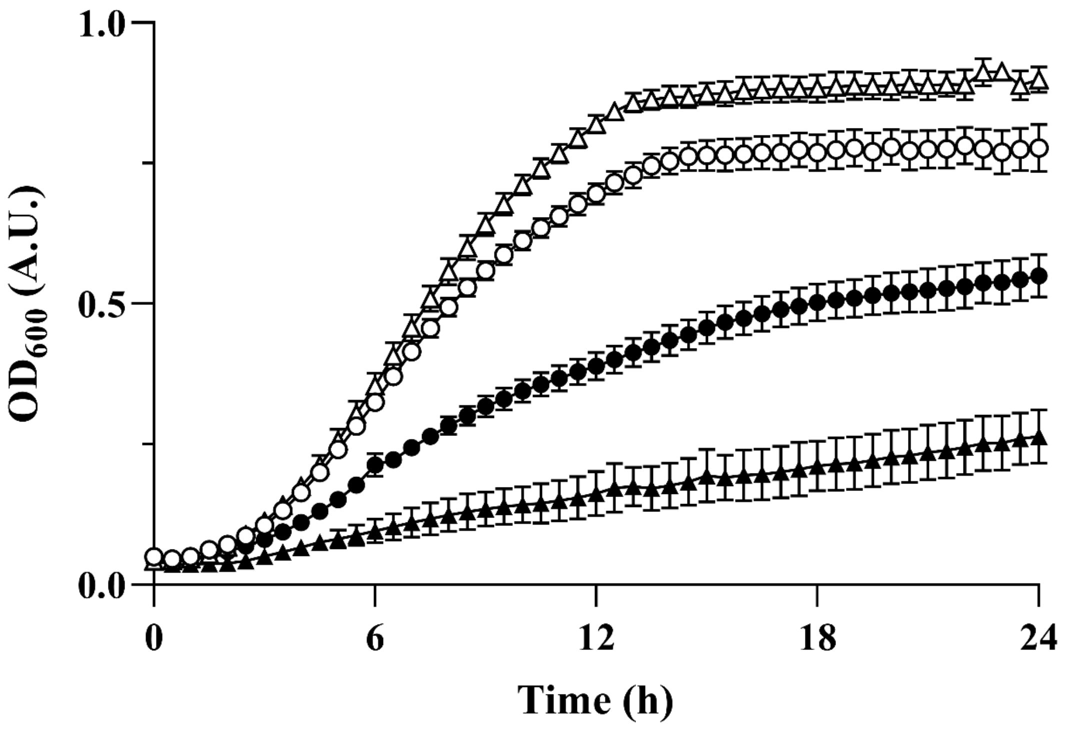

Figure S1: Growth of ZM4 BX and ZM4 BXL strains in glucose or glucose plus xylose: open triangles: ZM4 BXL on glucose; open circles: ZM4 BX on glucose; closed triangles: ZM4 BXL in glucose and xylose. closed circles: ZM4 BX on glucose and xylose.

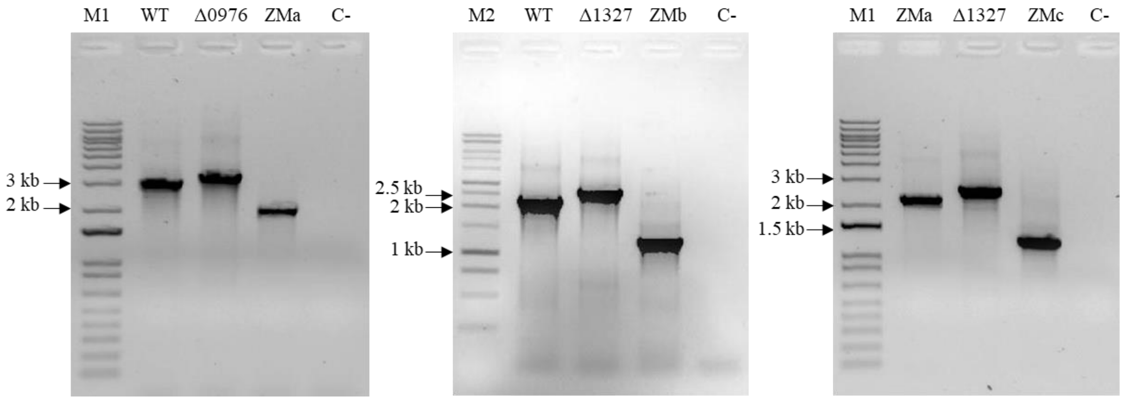

Figure S2: Deletion of genes ZMO0976 and ZMO1237 in *Z. mobilis*. After transformation, gene deletion and marker excision were assessed by PCR with specific primers. A: Deletion of ZMO0976 (confirmation primers U-ZMO0976-F/D-ZMO0976-R). M1: 1 kb Plus DNA Ladder (Invitrogen), WT: wild-type ZMO0976 (~3 kb); Δ0976: ΔZMO0976::*Sp*^{R} (~3.3 kb); ZMa: ΔZMO0976 (~2.1 kb). B: Deletion of ZMO1237 (confirmation primers Cldh-F/Cldh-R). M2: 1 kb DNA Ladder (Promega); WT: wild-type ZMO1237 (~2.2 kb); Δ1237: ΔZMO1237::*Sp*^{R} (~2.5 kb); ZMb: ΔZMO1237 (~1.3 kb). C: Deletion of ZMO1237 in ZMa strain. M1: 1 kb Plus DNA Ladder (Invitrogen); ZMa: ΔZMO0976 - wild-type ZMO1237 (~2.2 kb); Δ1237: ΔZMO0976, ΔZMO1237::*Sp*^{R} (~ 2.5 kb, primers Cldh-F/Cldh-R); ZMc: ΔZMO0976, ΔZMO1237 (~ 1.3 kb, primers Cldh-F/Cldh-R). *Sp*^{R} : spectinomycin resistance cassette. C- : reaction control.

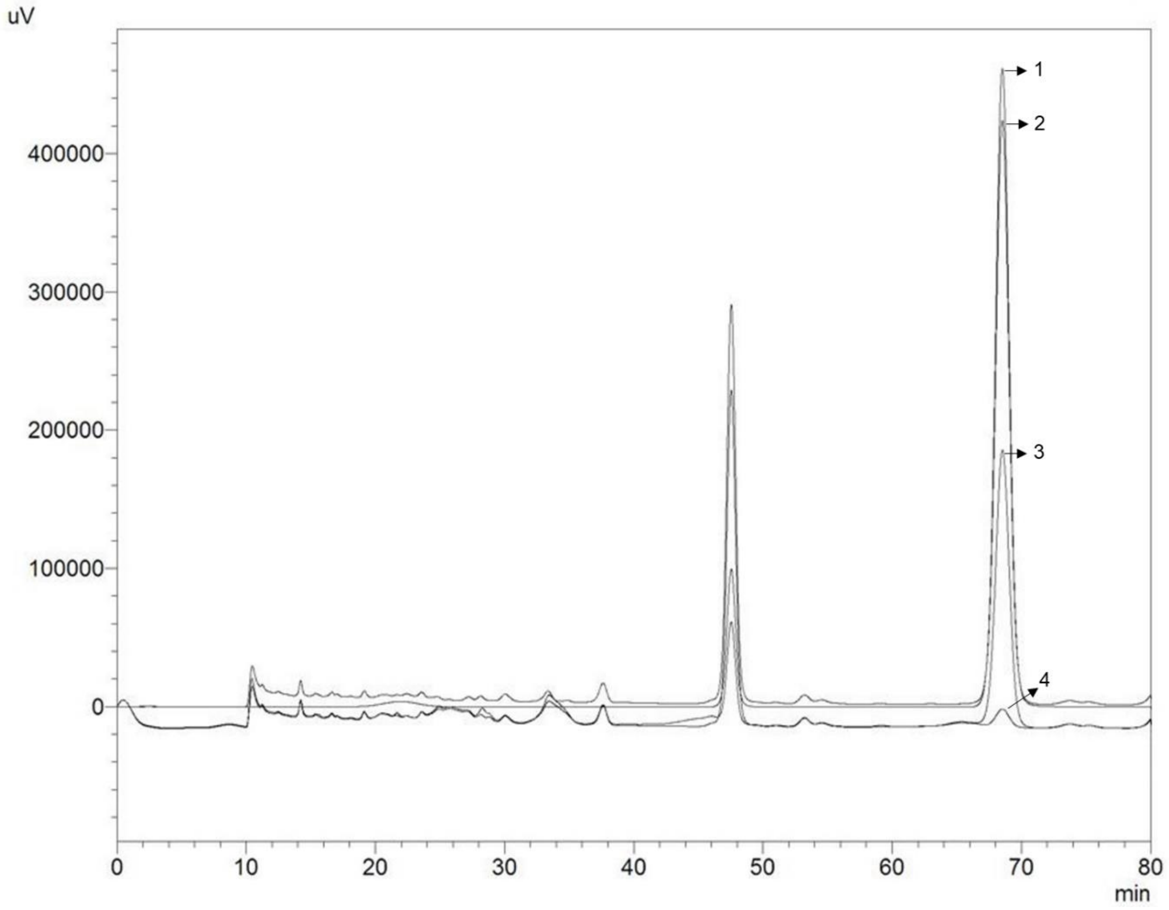

Figure S3: Stacked chromatograms showing the decrease in furfural peak (retention time ~70 min). Standard containing furfural (1); fermentation sample at time 0 h (2); fermentation sample after 4 h (3); fermentation sample after 20h (4).

Table S1: Primers used for amplification of XDH genes, construction of deletion cassettes, knockout confirmation and Cre recombinase amplification.

Primer	Sequence (5'→ 3')	Use
XDH_BS-F	ACATAGTGTTTTGAATATATGGAGTAAGCATATGATG TCCGGTGCTGTTTACCC	Amplification of gene BS
XDH_BS-R	GCCTTAAGCTCTAAGTTTATTTAAAAAAGATCTTTATC TCCAACCAGCGTCAACGA	
XDH_HL-F	AGTGTTTTGAATATATGGAGTAAGCATATGATGACTC ACCCATCTGCTAGATAC	Amplification of gene HL
XDH_HL-R	CTTAAGCTCTAAGTTTATTTAAAAAAGATCTTTAACCC CAACCACCATCAACTG	
XDH_CC-F	ACATAGTGTTTTGAATATATGGAGTAAGCATATGATG TCCTCTGCTATCTACCCATCC	Amplification of gene CC
XDH_CC-R	TCGCCTTAAGCTCTAAGTTTATTTAAAAAAGATCTTTA TCTCCAACCAGCGTCAATCC	
XDH_AP-F	ACATAGTGTTTTGAATATATGGAGTAAGCATATGATG GTTAACGCTGGTAACAGAACTGG	Amplification of gene AP
XDH_AP-R	TCGCCTTAAGCTCTAAGTTTATTTAAAAAAGATCTTTA AACCCAACCACCGTCAACGATG	
XDH_BX-F	ACATAGTGTTTTGAATATATGGAGTAAGCATATGATG TACTTGTTGTCCTACCCAGAGC	Amplification of gene BX
XDH_BX-R	TCGCCTTAAGCTCTAAGTTTATTTAAAAAAGATCTTTA TTCACCGTACCAACCAGCGTC	
XDH_TR-F	ACATAGTGTTTTGAATATATGGAGTAAGCATATGATG GCTTCTGGTAACCCATAC	Amplification of gene TR
XDH_TR-R	TCGCCTTAAGCTCTAAGTTTATTTAAAAAAGATCTTTA CTGGTTACCAGTGTTCAATG	
XDH_TM-F	ACATAGTGTTTTGAATATATGGAGTAAGCATATGATG GCTCCATACACTGCTAAG	Amplification of gene TM
XDH_TM-R	TCGCCTTAAGCTCTAAGTTTATTTAAAAAAGATCTTTA CTTTCTACCAGTGTTCAATGG	
UP-pV-F	CTATAGGGCGAATTGGGTACCCGCCATCCAAAAAATG G	Amplification of 5´ upstream region of ZMO0976
UP-Res-R	ACGAAGTTATCAGAAACTCTCCACATTTTTTAAAAAA G	
Res-UP-F	AGAGTTTCTGATAACTTCGTATAATGTATGCTATACG	Amplification of spectinomycin marker for ZMO0976 deletion cassette
Res-DW-R	ATATCCGCTCGGATCCATAACTTCGTATAGCATACATT ATACG	
DW-Res-F	ACGAAGTTATGGATCCGAGCGGATATAAAACAAGC	Amplification of 3´ downstream region of ZMO0976
DW-pV-R	AGGGAACAAAAGCTGGAGCTCTAATATGTTGGCTTGG GTG	
UPldh-F	GCGCGCGTAATACGACTCACTATAGGGCGAATTGGGT ACCGAAAGATCGTTACTTCCTTA	Amplification of 5´ upstream region of ZMO1237
UPldh-R	CATGATATAACTTCGTATAGCATACATTATACGAAGTT ATAGACACCCTCTTGAAAAG	
Resldh-F	CATATCTTTTTTCTTCAAAAAACTTTTCAAGAGGGTGT CTATAACTTCGTATAATGTATGCTATAC	Amplification of spectinomycin marker for ZMO1237 deletion cassette
Spcldh-R	GATCATTATGATCTTCAAAAGGTTAATTGTCGCTTGTC TAATAACTTCGTATAGCATACA	
DWldh_Spc-F	AAGGATATAACTTCGTATAATGTATGCTATACGAAGT TATTAGACAAGCGACAATTAACC	Amplification of 3´ downstream region of ZMO1237
Dwldh-R	CGCAATTAACCCTCACTAAAGGGAACAAAAGCTGGAG CTCAAAATACGCCTAAAAAAGTT	
U-ZMO0976-F	CTATCAGTCGTCCCTTATATGGTCTP	Confirmation of ZMO0976 deletion
D-ZMO0976-R	CTCCTTTTGGGTGCGGCG	
Cldh-F	TCGTTAAGCATCTGTCGAAA	Confirmation of ZMO1237 deletion
Cldh-R	GATTACGATCCAGAAAGTTATAAAAAC	
Cre-F	AGATCTCTAATCGCCATCTTCCAGCAG	Amplification of Cre recombinase gene
Cre-R	CATATGATGTCCAATTTACTGACCGTACACCAAAT	
